# Surface Functionalization and *Escherichia coli* Detection Using Surface-Enhanced Raman Spectroscopy Driven by Functional Organic Polymer/Gold Nanofilm-Based Microfluidic Chip

**DOI:** 10.3390/bios13120994

**Published:** 2023-11-21

**Authors:** Hugo Cortes-Cano, Lilian Iraís Olvera, Emilia M. Méndez-Aguilar, Beatriz Liliana España-Sánchez, Luis Gerardo Arriaga, Goldie Oza, José Herrera-Celis

**Affiliations:** 1 Dirección de Ciencia, Centro de Investigación y Desarrollo Tecnológico en Electroquímica, Parque Tecnológico Querétaro S/N, Sanfandila, Querétaro 76703, Pedro Escobedo, Mexico; hgcc_1312@hotmail.com (H.C.-C.); lespana@cideteq.mx (B.L.E.-S.); larriaga@cideteq.mx (L.G.A.); goza@cideteq.mx (G.O.); 2 Instituto de Investigaciones en Materiales, Universidad Nacional Autónoma de México, Apartado Postal 70-360, CU, Coyoacán, Ciudad de Mexico 04510, Mexico; lolvera@materiales.unam.mx; 3 Wexbooks, Colegio Wexford, Arco del Triunfo No.7, El Pueblito 76903, Maravillas, Mexico; em.mendezaguilar@gmail.com

**Keywords:** surface-enhanced Raman spectroscopy, *Escherichia coli*, microfluidic prototype, surface functionalization, microorganism detection, functional organic polymer, simulation, power enhancement factor

## Abstract

In this work, a microfluidic prototype based on polymeric materials was developed to monitor surface processes using surface-enhanced Raman spectroscopy (SERS), keeping the reagents free of environmental contamination. The prototype was fabricated on poly(methyl methacrylic acid) (PMMA). A micrometric membrane of a functional organic polymer (FOP) based on *p*-terphenyl and bromopyruvic acid monomers was formed on the PMMA surface to promote the formation of metal nanoclusters. Au nanosized film was deposited on the FOP membrane to give rise to the SERS effect. A microchannel was formed on another piece of PMMA using micromachining. A representative 3D model of the prototype layer arrangement was built and simulated in COMSOL Multiphysics^®^ to approximate the electric field distribution and calculate the power enhancement factor as the Au film changes over time. The fabrication process was characterized using UV–visible and Raman spectroscopies and XPS. The prototype was tested using a Raman microscope and liquid solutions of cysteamine and *Escherichia coli* (*E. coli*). The simulation results demonstrated that the morphological characteristics of the Au layer give rise to the SERS effect, and the power enhancement factor reaches values as high as 8.8 × 10^5^ on the FOP surface. The characterization results showed the formation of the FOP and the Au film on PMMA and the surface functionalization with amine groups. The Raman spectra of the prototype showed temporal evolution as different compounds were deposited on the upper wall of the microchannel. Characteristic peaks associated with these compounds were detected with continuous monitoring over time. This prototype offers many benefits for applications like monitoring biological processes. Some advantages include timely surface evaluation while avoiding environmental harm, decreased use of reagents and samples, minimal interference with the process by measuring, and detecting microorganisms in just 1 h, as demonstrated with the *E. coli* sample.

## 1. Introduction

Infectious diseases have gained relevant interest due to the increased number of deaths caused by pathogens. Among the primary pathogens causing these deaths, the Shiga toxin-producing strains of *Escherichia coli* (*E. coli*) stand out. According to Murray et al., around 200,000–800,000 human deaths globally were attributed to and associated with *E. coli* infections [1]. Recent reports show that the exponentially increasing death trend is due to increased antibiotic resistance by microorganisms [2,3]. The WHO reports that *E. coli* resistance to ciprofloxacin has risen from 8.4% to 92.9% and that 47% of bloodstream infections are due to *E. coli* resistance to third-generation cephalosporins [4]. In addition, more than 20% of the cases registered in the Global Antimicrobial Resistance and Use Surveillance System (GLASS) in 2020 referred to *E. coli* infections in the bloodstream and urinary tract which exhibited resistance to ampicillin and co-trimoxazole [5]. Given this situation, developing new methods and devices for rapidly detecting and monitoring microorganisms is a priority task. Until now, conventional and emerging detection methods such as plate counting, polymerase chain reaction (PCR), and enzyme-linked immunosorbent assay (ELISA) have been used to achieve this purpose [6]. However, these methods have a long sample processing time (several days) and require sophisticated laboratory equipment. In this sense, new techniques and devices with integrated microfluidics have already been developed to reduce the processing time [7,8,9,10].

Many methods and devices have been developed to detect, quantify, and identify *E. coli*. Ionescu conducted a review of electrochemical, optical, and acoustic biosensors created for the detection of *E. coli* [11]. Some optical and acoustic biosensors have the following performance: low detection limits and label-free, real-time detection capabilities, as well as quick and steady reaction times. He also integrated biosensors into microfluidic systems, thus developing a paper microfluidic device to be used in conjunction with smartphone technology to identify *E. coli* in water without functionalization with antibodies. However, this approach is limited by its inability to distinguish between similar bacterial species, such as *E. coli* and *Salmonella* spp. [11]. Subsequently, a survey of the techniques employed to detect *E. coli* bacteria was conducted by Nurliyana et al. [6]. They analyzed conventional methods such as multiple-tube fermentation, plate count enumeration, membrane filtration, and emerging techniques such as PCR and ELISA. They also found that these methods take a longer time for sample processing, which is one of the significant issues in developing quick tests for detecting *E. coli*.

Biosensors seem more exciting due to their smaller size, minimal and lower time-consuming sample preparation, portability, and in situ work. Under these considerations, several approaches have been achieved in recent years using electrochemical techniques [12,13,14]. For example, Hannah et al. proposed platforms based on screen-printed electrodes to monitor *E. coli* growth and evaluate its response to streptomycin, an antibiotic used to treat tuberculosis, using single-frequency electrochemical impedance. Regarding detection, conventional techniques such as chronoamperometry [12], electrochemical impedance spectroscopy [13], cyclic voltammetry [13,14], and square-wave voltammetry [14] have been tested using bacterial attachment [12,13] to electrode surfaces and bifunctional nanoparticles [14] as a mechanism to hinder a redox reaction. The results demonstrate that it is possible to identify bacteria in a shorter time than with PCR and ELISA [13,14]. However, these methods are indirect and still require complex procedures of sample or surface preparation to ensure selectivity.

Surface-enhanced Raman spectroscopy (SERS) stands out for being label-free, non-invasive, highly sensitive, and capable of operating in real time, among others. Mosier-Boss presented a review of SERS applied to the characterization and detection of bacteria [15]. She focused on studying interactions between Au/Ag nanoparticles (NPs) and bacteria and how these interactions impact bacterial activity. The study was carried out under three schemes: bacteria mixed with a Ag colloid, bacteria on an active SERS substrate, and bacteria and Ag colloid mixed and placed on a substrate. As a result, she found that, in addition to secretion information, the coating of the bacterium surface with Ag/Au NPs gives information about the cell wall composition. Furthermore, using 514 and 633 nm wavelength lasers, she demonstrated that the kind of substrate employed and the excitation wavelength could affect the spectrum [15]. Consequently, the choice of these elements is critical. On the other hand, SERS was presented by Su et al. as a technique for identifying and searching bacteria [16]. They used a Au colloid mixed with the bacteria to apply the SERS technique, obtaining precise fingerprint spectra for *E. coli* and *Salmonella typhimurium*. However, it implies pre-processing the sample and post-processing data with principal component analysis (PCA) and hierarchical cluster analysis (HCA) to classify bacteria [10,16,17,18]. SERS tests on bacteria conducted by Witkowska et al. demonstrated that this technique has a helpful approach for bacterial samples and may be used as an alternative method in compliance with the requirements of the International Organization for Standardization (ISO) [10]. With direct SERS analysis of bacterial colonies grown on selective medium agar, Witkowska et al. reduced the analysis time from 6 to 2 days. Adding PCA calculations, they achieved remarkable results in bacteria-contaminated food analysis with 98% accuracy in just 48 h [10].

Several works have demonstrated the convenience of microfluidic prototypes in applying the SERS technique [7,8,9]. Wang et al. observed that combining microfluidic devices with SERS detection results in a simpler, miniaturized, and adequate instrumentation for detecting and characterizing chemical and biological analytes in small sample volumes [8]. Thus, high sensitivity is provided by this innovative SERS pathogen detection method. In this instance, no washing was involved in obtaining the stacked spectra. Consequently, successfully identifying and isolating bacteria in water samples without additional washing stages or secondary antibody reporting could be possible. Thus, this platform outperformed ELISA kits in terms of efficiency, detection time, and cost [8]. An integrated platform of a plasmonic bacterium on a nanoporous glass membrane was created by Whang et al. employing hydrodynamic trapping for bacterial enrichment and significant signal amplification [9]. According to fluid dynamics simulations, enrichment occurs when bacteria are caught in the nanopore during filtering. The platform can offer details on microbial surface expression correlated with the effects of emerging pathogen genomes [9]. Srivastava et al. created a nanobiosensor device to precisely and quantitatively detect two *E. coli* strains [7]. This sensor could pick up to 1.5 × 10^2^ CFU/mL (CFU, colony-forming units). Additionally, the sensor was designed for samples of 10 μL volume.

Some improvements to the SERS technique have also been achieved by incorporating NPs and novel detection mechanisms. Chisanga et al. showed a novel application strategy of SERS, using it together with isotopic labeling for the quantitative detection and classification of *E. coli* bacteria [19]. Through isotope labeling and SERS, they obtained information on the function of the organism and its differentiation within complex communities. A dual biological sensor built on NPs was developed by Boca et al. [20]. This kind of sensor can act as a chemoresistor and a SERS substrate. They identified the species that modify the electrical resistance via SERS measurements. These findings can be applied to create sophisticated sensors, including their incorporation into microfluidic devices. Yang et al. presented a label-free and SERS-based method for detecting *E. coli* using incubation with Ag colloids and demonstrated that greater Raman intensities could be achieved when NPs and cells are closer [18]. According to Gao et al., acquiring direct SERS spectra of microorganisms is linked to modifying Raman marker molecules, which must undergo a complex modification process before bacterial detection [21]. Therefore, they created NPs using an aptamer template to eliminate these conditioning variables. This strategy allowed them to create unique SERS spectra for bacteria whose survival depends on the distinct identification of aptamers and bacteria. Consequently, single-cell identification of *S. aureus* is made simple with the SERS mapping approach. Therefore, using a matching aptamer, this SERS bioassay may be widely utilized to identify harmful bacteria or cancerous cells [21].

Based on the reports discussed in the above paragraphs, this work presents the design, manufacture, and testing of a microfluidic prototype with applied nanotechnology based on polymeric materials to detect *E. coli* with SERS. The prototype is mainly based on a gold nanofilm deposited by e-beam evaporation, which is assisted by the surface chemistry of a functional organic polymer to give rise to metal nanoclusters with SERS-friendly properties. Unlike previous developments, this work shows that it is possible to monitor functionalization processes and *E. coli* attachment using the same setting. The prototype achieves *E. coli* detection without requiring complex SERS platform preparation processes, bacterial pretreatments, or additional statistical analysis, which gives it manufacturing and application advantages. This opens the window to the development of new prototypes structurally based on polymeric materials for detection by SERS. To clarify the different aspects, the sections are organized as follows: The first section shows the design and manufacturing aspects of the prototype, including simulations carried out in COMSOL Multiphysics^®^ 5.6 (COMSOL). The following section addresses the methodology for prototype monitoring and detection tests. Finally, the results and discussion emphasize the proposal’s viability for monitoring and detecting microorganisms.

## 2. Design and Simulation in COMSOL

### 2.1. Design Considerations

**Figure 1:**
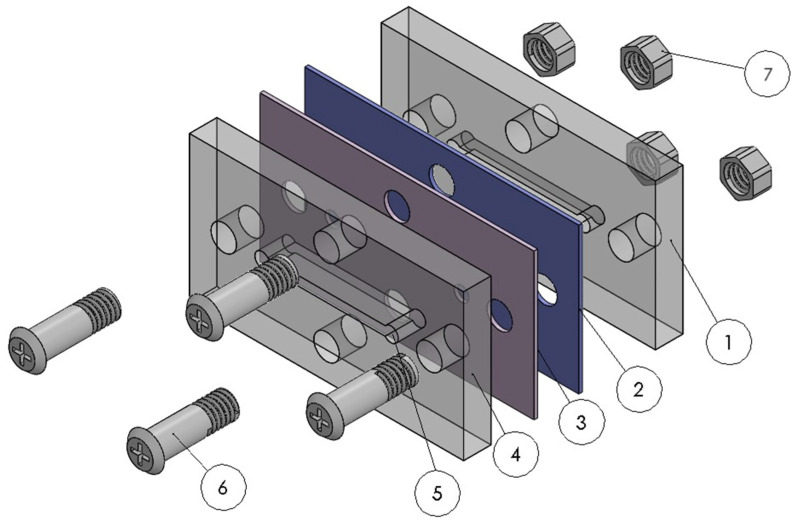
Illustration of the microfluidic prototype by components. (1) PMMA bottom cover plate with microchannel; (2) 5 nm Au layer; (3) 1 µm of functional organic polymer; (4) PMMA top cover plate with polymeric suspended membrane; (5) inlet/outlet ports of the microchannel; (6) 1.4 mm diameter bolts; and (7) nuts. PMMA: poly(methyl methacrylic acid); Au: gold.

The proposed prototype is shown in Figure 1 and comprises an arrangement of cover plates and layers based on polymeric materials. Both the top and bottom cover plates are composed of poly(methyl methacrylic acid) (PMMA), a polymer that is mechanically stable, transparent to visible light, biocompatible, and has low autofluorescence. In addition, PMMA is compatible with microfabrication processes in the microfluidic industry. The middle layers consist of a functional organic polymer (FOP) and a nanometric gold (Au) film, which is responsible for the SERS effect. The radiation initially passes through the upper PMMA cover plate, which contains a cavity that aligns with the microfluidic channel created in the lower PMMA cover plate. This results in a rigid PMMA membrane that avoids deformations or deflections, while also being thin enough to minimize its optical effects during the application of the SERS technique.

Aspects such as the sensing area, the volume of the sample, and the coupling with the objective of the Raman microscope were considered in the geometric parameters of the prototype. Regarding the dimensions, each piece of PMMA is 1.5 mm in thickness, 16 mm in length, and 8 mm in width. The thicknesses of the FOP and Au films are 1 µm and 5 nm, respectively. The microchannel built in the bottom PMMA cover plate is an orthoedron of 6.25 mm in length, 1 mm in width, and 800 µm in height, resulting in a volume of 5 µL and a sensing surface area of 6.25 mm^2^. Considering the mean dimensions of the *E. coli* bacterium (2 µm in length and 0.5 µm in diameter), the sensing area can house 1 × 10^8^ CFU/mL.

### 2.2. Simulation Setup

**Figure 2:**
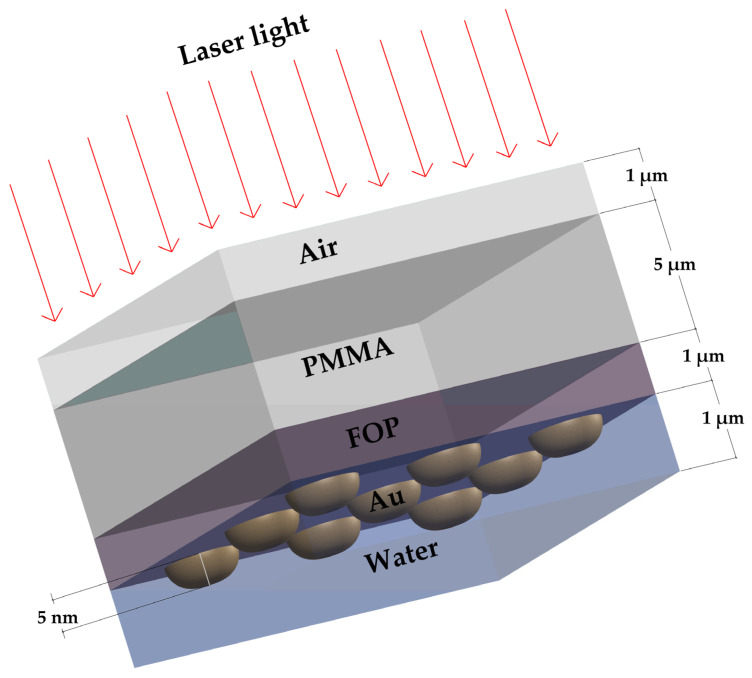
A 3D representation of the proposed microfluidic prototype. PMMA: poly(methyl methacrylic acid); FOP: film of functional organic polymer based on *p*-terphenyl and bromopyruvic acid monomers; Au: gold thin film.

The 3D model of the layer arrangement including water was created in COMSOL using the model assistant [22]. Water was chosen as the liquid medium commonly used for bacterial detection. From the wave optics module of COMSOL, the electromagnetic waves–frequency domain and the frequency domain were selected as the physical interface and study type, respectively. The size of the 3D model was varied according to the results reported by Hernández-Cruz et al. for 5 nm post-deposited Au films [23]. They reported that the Au layer modifies its morphology very slowly (the process takes more than 20 days), and linear relationships of the width and spacing of the Au nanoclusters versus time were assumed [23]. The layer ordering of the 3D model, from top to bottom, was as follows: air (1 µm thick), PMMA membrane (5 µm thick), FOP film (1 µm thick), Au film (5 nm thick), and water (1 µm thick). Figure 2 illustrates the layer arrangement.

The air layer mimics the gap between the prototype and the objective of the microscope. The Au film was modeled using flattened nano-hemispheres that were evenly spaced apart from each other. Floquet periodicity was set for the four side walls of the unit cell assuming the model periodicity along the x and y axes. A free triangular mesh was defined on the surface of the nano-hemispheres with the maximum element size being 10 times the minimum size, while a free tetrahedra mesh was defined for the rest of the design with a maximum element size of 100 times the minimum size. The minimum size scan (MeshSize parameter) in all simulations was between 0.1 and 1.0 nm. The power enhancement factor (PEF, (|*E*|/|*E*_0_|)^2^) distributions in the xy, xz, and yz planes around the nano-hemisphere were graphically depicted, and the maximum and average SERS enhancement factors (EFs) were calculated following the |*E*|^4^-approximation [24]. The software simulates an electromagnetic wave of frequency ω propagating through the layers from the input port (air) to the output port (water) in the z direction and with the electric field (***E***) perpendicular to the direction of propagation. In terms of mathematics, the software resolves the following partial differential equation in the frequency domain [25]:

(1) $$\nabla \times {\mu_{r}}^{-1}\left(\nabla \times E\right)={k_{0}}^{2}\left(\varepsilon_{r}-j\sigma /\omega \varepsilon_{0}\right)E,$$

where *k*_0_ is the wave number for propagation in free space, *ε*_0_ is the permittivity of free space, *j* is the imaginary number, and *µ_r_*, *ε_r_*, and *σ* are the relative permeability, relative permittivity, and conductivity of the medium where the wave propagates, respectively.

From an electric field applied to the input port (***E_in_***):

(2) $$E_{in}=E_{0}e^{-jk_{z}z},$$

where *E*_0_ is the magnitude of the applied electric field, the software approximates a solution to Equation (1) in the space assuming *μ_r_* = 1 and *σ* = 0. Finally, Table 1 lists the real and imaginary parts of the complex refractive index used in the simulation for each material of the 3D model.

## 3. Materials and Methods

### 3.1. FOP Synthesis

The polymer synthesis was carried out by a polyhydroxyalkylation reaction of bromopyruvic acid (Sigma-Aldrich, Toluca, Mexico, #16490, 0.88 g) with the multi-ring aromatic non-activated hydrocarbon *p*-terphenyl (Sigma-Aldrich, #T3203, 1.21 g) at 25 °C. A mixture of these monomers in dichloromethane was stirred under an ice bath; then, trifluoromethanesulfonic acid (Sigma-Aldrich, Toluca, Mexico, #347817, TFSA) as a catalyst was added, and the reaction continued until an increase in viscosity was observed. Then, the mixture was poured into anhydrous methanol (Sigma-Aldrich, Toluca, Mexico, #322415), obtaining a white polymer, which was filtered off and washed several times with hot methanol. Finally, the FOP was allowed to dry for 24 h at room temperature in a sealed container.

### 3.2. Prototype Manufacturing

A 4-inch PMMA wafer was used to fabricate the prototype supports. The pieces were micromachined using a micro-CNC Optimac5 (micro-CNC) manufactured by AREMAC Polymer. End mill bits of 1 mm (Kyocera, Kyoto, Japan, #1610-0394.118) were used for micromachining of the cavity, the microchannel, and to cut the pieces, whereas drill bits of 0.8 mm (Kyocera, Kyoto, Japan, #105-0315.400) and 1.4 mm (Kyocera, Kyoto, Japan, #105-0551.400) were used to open the inlet/outlet holes for fluids and holes for the screws, respectively. Before the deposition of the FOP film, the PMMA wafer was cleaned sequentially in isopropyl alcohol (Sigma-Aldrich, Toluca, Mexico, #190764) and deionized water, in both cases, for 10 min under sonication. Finally, the wafer was dried under nitrogen flow.

To deposit the FOP film, the synthesized FOP was first dissolved at 6% *w*/*v* in cyclohexanone (Sigma-Aldrich, Toluca, Mexico, #C102180). Next, the deposition process was carried out by spin coating at 2000 rpm for 5 min in an SCS Spin Coater Series manufactured by Specialty Coating Systems Inc. Next, one side of the wafer was covered with the FOP solution using a 5 mL syringe connected to a 0.45 μm filter (Biomed Scientific, Hong Kong, China, #SFPVDF025045) to avoid microparticle deposition. Finally, the wafer was annealed at 80 °C for 1 min on a hot plate to evaporate the solvent.

After FOP film deposition, the wafer was installed in the micro-CNC compartment and micromachined, obtaining several pieces ④ (see Figure 1). Under the same procedure, another PMMA wafer without FOP film was micromachined to obtain several pieces ①. The 5 nm thick Au film was deposited on the micromachined pieces ④ using a D18 e-beam evaporator manufactured by Intercovamex. Based on the research conducted by Milazzo et al. [30], the Au surface underwent treatment for 5 s with a solution of hydrofluoric acid (Sigma-Aldrich, #30107, HF) in deionized water at a ratio of 7:1 (H_2_O:HF). Afterwards, the pieces were rinsed with deionized water and dried with nitrogen flow. Finally, the micromachined and surface-modified pieces were assembled using screws and nuts. The prototype was checked for leaks by injecting a dye into the microchannel with a syringe.

### 3.3. Cysteamine Functionalization

As an initial test for detection capabilities of the prototype, the Au surface was functionalized with thiol groups. A 20 mM cysteamine (Cys) solution was prepared by dissolving cysteamine hydrochloride (Sigma-Aldrich, Toluca, Mexico, #M6500) in ethanol (J.T. Baker, Philadelphia, PA, USA, #9000-03) for 1 h under magnetic stirring at room temperature. Before cysteamine functionalization, the samples were irradiated with UV light for 15 min to activate the Au surface [31]. This study used PMMA/FOP/Au arrays and prototypes treated with UV light. The arrays were immersed in the cysteamine solution for 1 h, followed by rinsing in deionized water and drying under nitrogen flow. In the case of prototypes, ethanol was injected initially into the microchannel to obtain a reference measurement. After removing ethanol, the cysteamine solution was injected into the microchannel. The functionalization process was monitored for 4 h. After that, the microchannel was cleaned by injecting and extracting ethanol sequentially (three times minimum).

### 3.4. Bacterium Detection Using As-Fabricated Prototype

The detection probe was carried out using the ATCC^®^ 25922^TM^ strain of *E. coli* bacteria cultivated in a Luria–Bertani (LB) medium. The concentration of *E. coli* in the LB medium obtained by measuring optical density (OD) at 600 nm was 3.2 × 10^8^ CFU/mL. First, the LB medium was injected into the microchannel to obtain a reference spectrum. After removing the LB medium from the microchannel, the *E. coli* culture was injected into the microchannel. The interaction of the *E. coli* bacteria with the upper wall of the microchannel was monitored for 1 h. Subsequently, the *E. coli* sample was extracted from the microchannel and cleaned by injecting and extracting deionized (DI) water several times. Finally, the spectrum with DI water in the microchannel was registered.

### 3.5. Measurements

The manufacturing process was tracked using UV–visible spectroscopy and profilometry. The result of the cysteamine functionalization process was determined with X-ray photoelectron spectroscopy (XPS), whereas the prototype was tested with Raman spectroscopy. A Dektak 6M profiler manufactured by Veeco (Oyster Bay, NY, USA) was used to measure the thicknesses of the films; the UV–visible spectrophotometer BK-UV1000 manufactured by BIOBASE (Wolfenbuettel, Germany) was used to register the UV–visible spectra of a PMMA substrate as the layers of FOP, Au, and cysteamine were deposited; the K-Alpha XPS system manufactured by Thermo Fisher Scientific was used to characterize the self-assembled monolayer after functionalization.

All SERS spectra were obtained using an XploRA One confocal micro-Raman spectrometer manufactured by HORIBA Scientific (Northampton, UK), coupled with an Olympus BX41 microscope with a Raman spectrometer mounted on top of a thermoelectrically cooled CCD detector. The measurement range was from 200 to 3200 cm^−1^; the light source was a class 3B of 785 nm and 90–100 mW. The laser beam was focused on a spot size of around 3.8 µm using a 10X objective lens. A grating of 1200 lines/mm with a slit of 100 μm was chosen, resulting in an average spectral resolution of 2.7 cm^−1^ in the spectral field of 150–1250 cm^−1^. Three accumulations were used for all measurements, with a total collecting time of 30 s. The LABSPEC 6 software was used to gather and analyze Raman spectra. The obtained Raman spectra were subjected to post-processing to provide findings with the least amount of fluorescence or noise, including baseline correction, spectra smoothing, and peak searching/fitting. Additionally, some spectra underwent a normalizing step when it was required.

## 4. Results

### 4.1. Simulation Results

**Figure 3:**
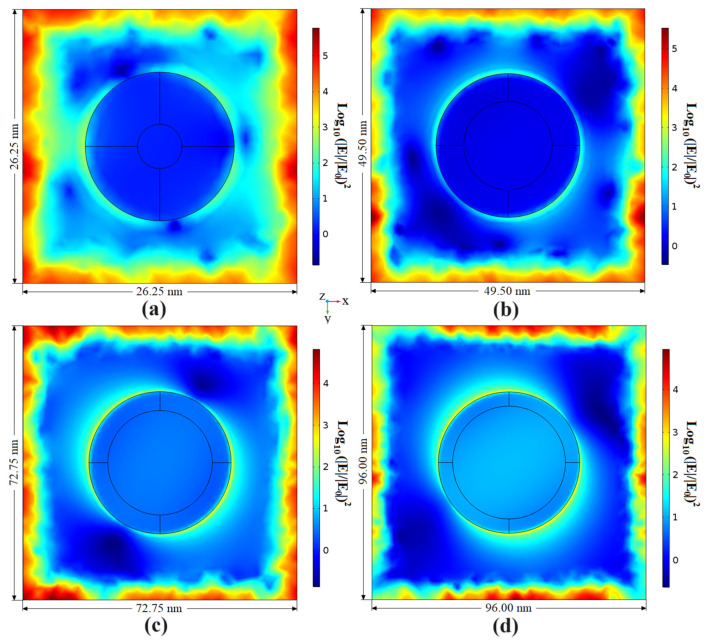
Distribution of the power enhancement factor ((|*E*|/|*E*_0_|)^2^) on the functional organic polymer surface obtained by simulation of the 3D model in COMSOL. The corresponding pairs of nano-hemisphere diameter and spacing were (14.25, 12.00), (26.00, 23.50), (37.75, 35.00), and (49.5, 46.5) in nanometers corresponding to (**a**) 6 (MeshSize = 0.6 nm), (**b**) 12 (MeshSize = 0.7 nm), (**c**) 18 (MeshSize = 0.3 nm), and (**d**) 24 (MeshSize = 0.5 nm) days. The circles represent the contours of the top and bottom surfaces of the nano-hemispheres.

**Figure 4:**
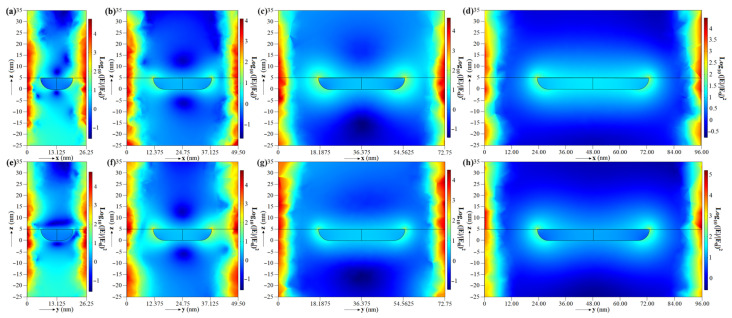
Distribution of the power enhancement factor ((|*E*|/|*E_0_*|)^2^) in the xz (top) and yz (bottom) planes within 30 nm above and below the nano-hemisphere. The pairs of graphs (**a**,**e**), (**b**,**f**), (**c**,**g**), and (**d**,**h**) correspond to the model simulated in COMSOL with nano-hemispheres after 6 (MeshSize = 0.6 nm), 12 (MeshSize = 0.7 nm), 18 (MeshSize = 0.3 nm), and 24 (MeshSize = 0.5 nm) days, respectively.

According to Hernández-Cruz et al., the Au film deposited on FOP material is transformed into nanoparticles over time, resulting in more extensive and more spaced nanoparticles daily [23]. Figure 3 and Figure 4 show the simulation results of the 3D model described in Section 2.2 considering 6, 12, 18, and 24 days of post-deposition time. According to Figure 3, the PEF is higher close to the nano-hemisphere and around the boundary of the 3D models in the xy plane at z = 5 nm (the FOP-Au interface). Figure 4 additionally shows that this electric field intensification at the boundaries of the nano-hemispheres extends along the *z* axis less than 5 nm above and below them. In contrast, the electric field intensification at the boundaries of the unit cell extends along the *z* axis until the boundaries of the graphs. It is also observed that as the size and gap of the nano-hemispheres are greater, the distribution of the electric field tends to be less intensive and more uniform. The average and maximum EFs for the planes in Figure 3 and Figure 4 are listed in Table 2. The local intensity of the electric field tends to decrease over time. The maximum (|*E*|/|*E*_0_|)^2^ varied from 8.8112 × 10^5^ after 6 days of post-deposition time to 3.2266 × 10^5^ after 24 days. These results show that it is possible to obtain the SERS effect with the proposed microfluidic prototype.

### 4.2. Manufacturing Step Tracking

**Figure 5:**
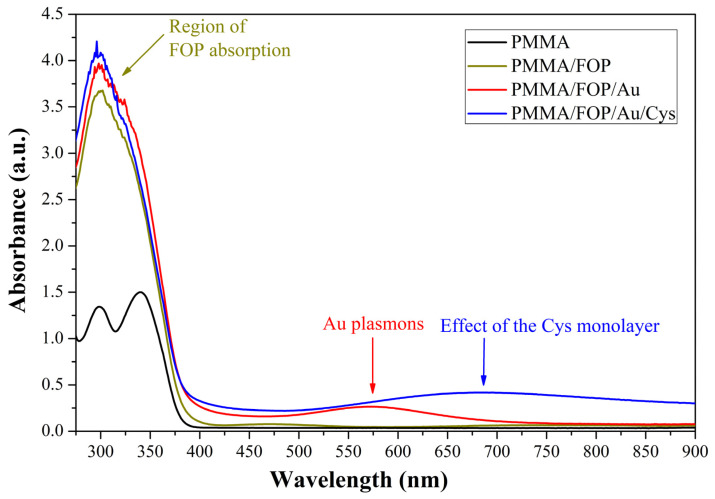
UV–vis spectra of the PMMA substrate as a reference and modified with the FOP, FOP/Au, and FOP/Au/Cys films. PMMA: poly(methyl methacrylic acid); FOP: film of functional organic polymer based on *p*-terphenyl and bromopyruvic acid monomers; Au: gold thin film; Cys: cysteamine.

Figure 5 shows the UV–visible spectra of the PMMA substrate as a reference and modified consecutively with the FOP, FOP/Au, and FOP/Au/Cys films. Spectra were captured in a wavelength range of 275–900 nm with a resolution of 1 nm. From these results, the FOP has an absorbance maximum between 275 and 350 nm. When the Au film is deposited, a relief appears in the range of 500 to 650 nm (absorption maxima at 573 nm). This relief is attributed to the formation of Au nanoclusters after film deposition. Regarding the spectrum corresponding to the functionalization with cysteamine, an increase in the absorption level from 600 to 900 nm was observed. These increases in absorption are attributed to the functionalization layer, which redistributes the Au plasmon resonance and shifts its maximum towards higher wavelengths, thus leading to the hyperchromic effect [32].

**Figure 6:**
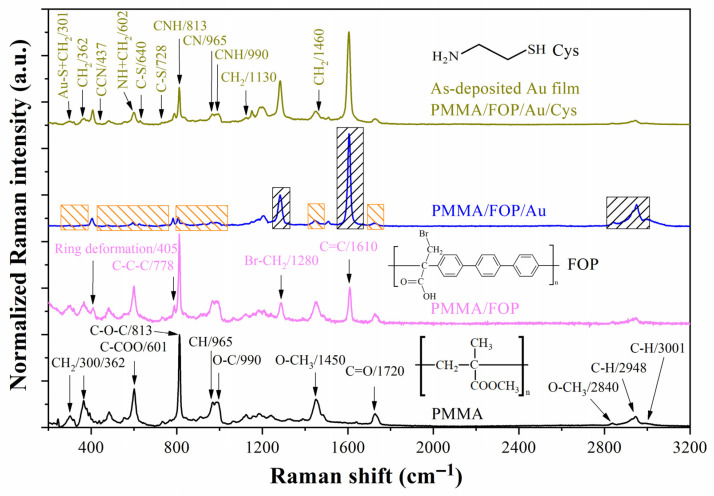
Raman spectra of the PMMA substrate and PMMA/FOP, PMMA/FOP/Au, and PMMA/FOP/Au/Cys layer arrays. PMMA: poly(methyl methacrylic acid); FOP: film of functional organic polymer based on *p*-terphenyl and bromopyruvic acid monomers; Au: gold thin film; Cys: cysteamine.

Figure 6 shows the evolution of the Raman spectrum as the different layers were deposited. PMMA presented molecular vibrations at 300, 362, 601, 813, 990, 1450, 1720, 2840, 2948, and 3001 cm^−1^ associated with its molecular groups (see the black trace in Figure 6) [33,34]. After FOP deposition, four peaks were added to the spectrum at 405, 778, 1280, and 1610 cm^−1^, corresponding to the deformation of the rings and the molecular groups C-C-C, Br-CH_2_, and C=C, respectively [33,35]. The PMMA-associated peaks were diminished after Au deposition (see orange boxes on the blue trace in Figure 6); in contrast, the FOP-associated peaks were intensified (see black boxes on the blue trace in Figure 6) as a result of the SERS effect of the Au film. After the cysteamine functionalization process on the as-deposited Au film, several peaks were added to the spectrum. The peaks at 301, 362, 602, 1130, and 1460 cm^−1^ were assigned at vibrational modes of methyl groups [33]. The Au-S and N-H bonds share the Raman shift of CH_2_ groups at 301 and 602 cm^−1^, respectively [36,37]. The vibrational modes of C-N-H chains appeared at 813 and 990 cm^−1^, whereas the vibrational modes of C-S bonds were assigned at the peaks at 640 and 728 cm^−1^ [33,38]. A slight peak at 437 cm^−1^ was attributed to the C-C-N chains of cysteamine [39]. As can be seen, some Raman peaks of PMMA and cysteamine overlap; however, the distinction between them was possible because the as-deposited Au film (without treatment in HF solution) reduces the propagation of the radiation wave behind the Au layer, decreasing the intensity of molecular vibrations of the PMMA substrate. On the other hand, the SERS effect amplifies the Raman signals corresponding to the compounds closer to the Au surface (the FOP film and cysteamine monolayer).

**Figure 7:**
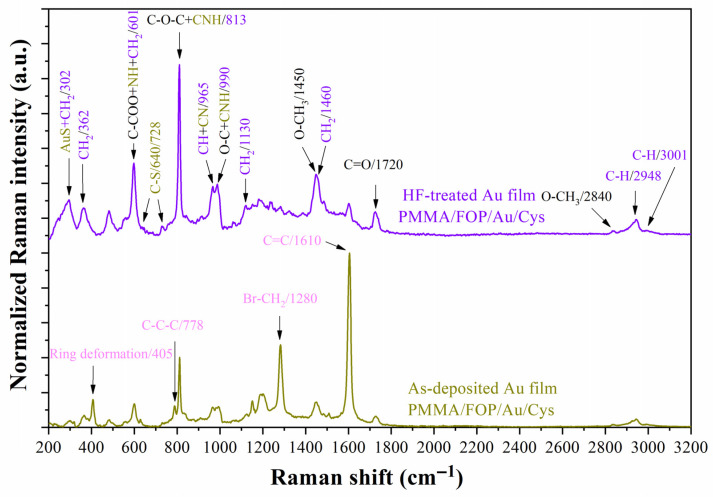
Raman spectra of the as-deposited (olive green line) and HF-treated (purple line) Au films deposited on PMMA/FOP substrates and functionalized with cysteamine (Cys). Black color assignments: Raman peaks characteristic of PMMA; pink color assignments: Raman peaks characteristic of functional organic polymer (FOP); olive green color assignments: Raman peaks characteristic of cysteamine; purple color assignments: Raman peaks shared by PMMA and cysteamine.

The HF treatment has two expected outcomes. Firstly, it cleans the surface before prototype assembly by removing the native oxide, and secondly, it accelerates the process of Au layer stabilization [29]. According to Schwartzkopf et al., during the Au film deposition, the lateral growth of Au clusters is stopped around 5.5 nm of thickness [40]. Since the Au film considered in this work is just 5 nm, Au-free nanospaces or weakly bonded Au atoms on the FOP surface are likely [41,42]. These Au atoms slowly join into clusters, resulting in a time-dependent process [23]. With HF treatment, the surface energy is reduced, and this process is accelerated. Figure 7 shows the Raman spectra of as-deposited and HF-treated Au films. According to these spectra, there is an increase in the Raman peaks associated with the PMMA substrate (black color assignment) and cysteamine functionalization monolayer (olive green color assignment) and a decrease in the peaks associated with the FOP film (pink color assignment). These results agree with the previous explanation (more spaced and bigger nanoclusters).

**Figure 8:**
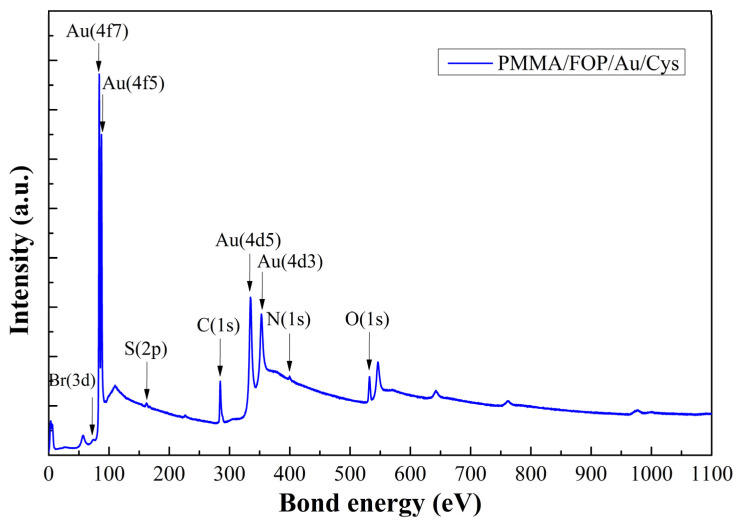
XPS spectrum of a PMMA/FOP/Au sample functionalized with cysteamine (Cys). PMMA: poly(methyl methacrylic acid) substrate; FOP: film of functional organic polymer based on *p*-terphenyl and bromopyruvic acid monomers; Au: gold thin film.

As verification of the functionalization process, a PMMA/FOP/Au sample treated with cysteamine solution was measured with XPS. According to Figure 8, the presence of Au is confirmed, with peaks at 84, 87, 335, and 352 eV [43]. In addition, the peaks detected at 400 eV and 162.1 eV for the N and S atoms, respectively, provide further evidence that the functionalization process was successful. These findings support the conclusions drawn from the Raman spectroscopy results [44]. Br and O atoms were also detected at 70 and 532 eV, respectively, which are related to the FOP film [45].

### 4.3. Monitoring of the Functionalization Process

**Figure 9:**
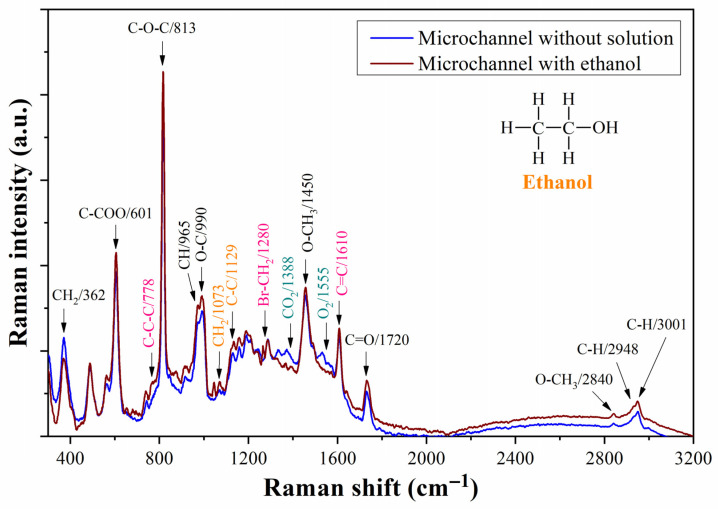
Raman spectra of the microfluidic prototype with and without ethanol in the microchannel. Black color assignments: Raman peaks characteristic of PMMA; pink color assignments: Raman peaks characteristic of functional organic polymer (FOP); dark cyan color assignments: Raman peaks characteristic of environmental air; orange color assignments: Raman peaks characteristic of ethanol.

To gain a better understanding of the functionalization process utilizing the microfluidic prototype, we conducted preliminary Raman measurements on the assembled prototype both with and without the presence of ethanol in the microfluidic channel. The main differences in the spectra of Figure 9 are due to the displacement of environmental air from the inner of the microchannel by ethanol. The peaks at 1335, 1388, and 1555 cm^−1^ corresponding to CO_2_ and O_2_ disappear in the case of the microchannel filled with ethanol [33,46]. Other peaks, such as those at 778, 990, 1073, and 1129 cm^−1^, increase their intensity due to the ethanol in the microchannel.

**Figure 10:**
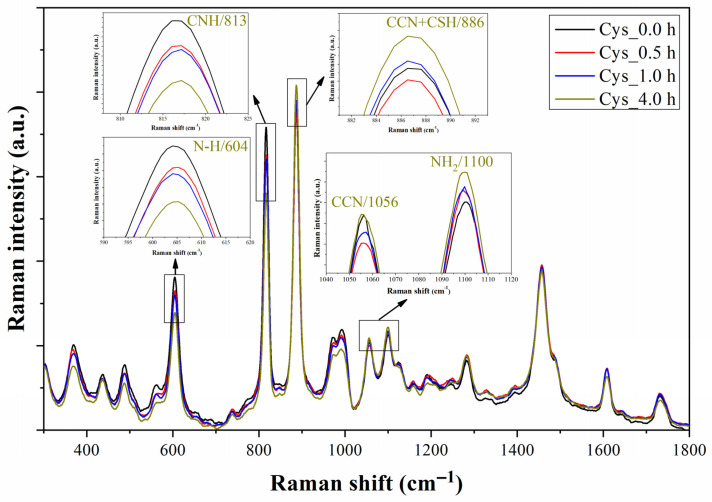
Raman spectra of the microfluidic prototype registered at 0, 0.5, 1, and 4 h after injecting cysteamine (Cys) solution in the microchannel. The most prominent characteristic Raman peaks of cysteamine at 604 (N-H), 813 (CNH), 886 (CCN + CSH), 1056 (CCN), and 1100 (NH_2_) cm^−1^ are detailed in the insets.

**Figure 11:**
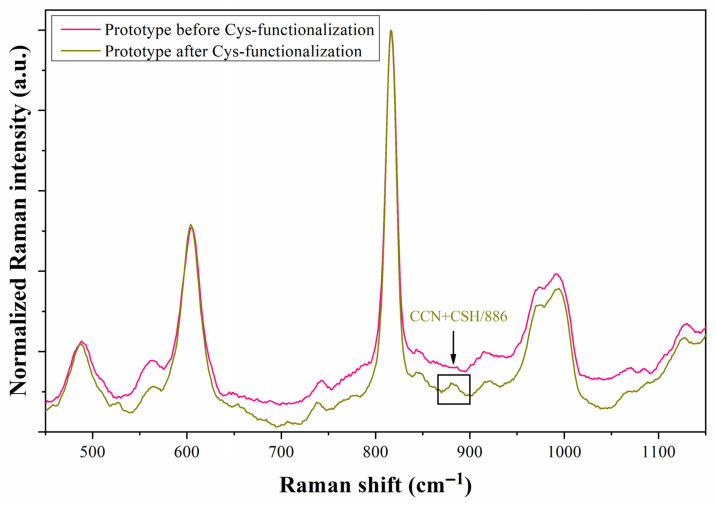
Raman spectra of the prototype before and after functionalization of the microchannel with cysteamine (Cys). The spectra were recorded under liquid-free microchannel conditions.

The Raman spectra of the prototype with cysteamine solution at different monitoring times, namely 0, 0.5, 1, and 4 h, are illustrated in Figure 10. The characteristic Raman peaks of cysteamine at 604 (N-H), 813 (CNH), 886 (CCN + CSH), 1056 (CCN), and 1100 (NH_2_) cm^−1^ are zoomed in the four insets of the graph, and their changes in intensity will be used to explain the evolution of the functionalization process [33,39]. The intensities of the Raman peaks corresponding to N-H and CNH decrease with time, whereas the primary peak corresponding to thiol groups increases its intensity, which agrees with the expected orientation of the molecule: the thiol groups in contact with the Au surface. This orientation favors the molecular vibration of the CCN chains and amino groups. Figure 11 shows the Raman spectrum of the prototype after extracting the cysteamine solution from the microchannel. After the functionalization test, the Raman spectrum of the prototype still shows a soft peak at 886 cm^−1^ compared to its original spectrum. Although this process did not reveal how the sulfur in the cysteamine molecule interacts with the Au surface of the microchannel’s upper wall (as in the previous section), it is worth noting that the prototype is capable of detecting the cysteamine monolayer, which is typically challenging to identify using conventional Raman and FTIR spectroscopies.

### 4.4. Bacterium Detection with As-Fabricated Prototype

**Figure 12:**
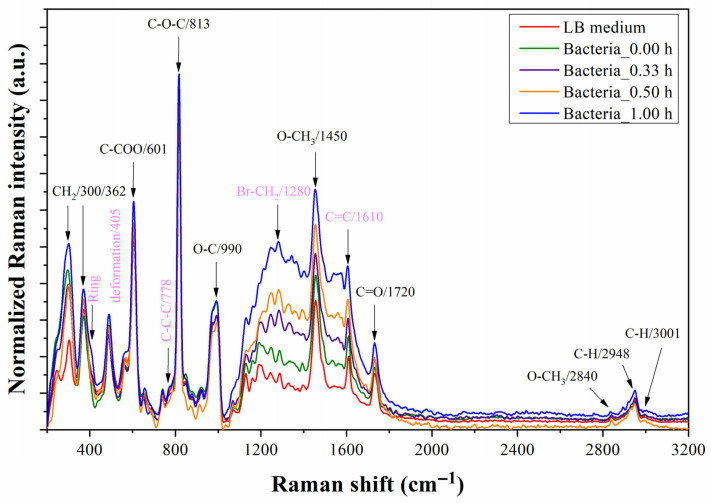
Raman spectra of the microfluidic prototype measured at 0, 0.33, 0.5, and 1 h after injecting *E. coli* culture in Luria–Bertani (LB) medium into the microchannel. The assigned Raman peaks correspond to the molecular vibrations of functional groups of the PMMA membrane and the functional organic polymer (FOP) film. Black color assignments: Raman peaks characteristic of PMMA; pink color assignments: Raman peaks characteristic of FOP.

**Figure 13:**
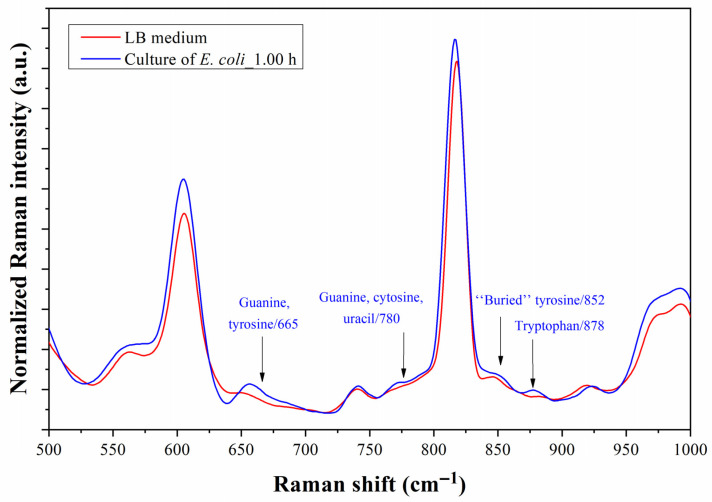
Raman spectra in the 500–1000 cm^−1^ band shift of the microfluidic prototype measured with LB medium before testing and *E. coli* culture after 1 h of testing. The assigned Raman peaks correspond to the molecular vibrations of functional groups of *E. coli*.

Figure 12 depicts the Raman spectra of the microfluidic prototype which was monitored for an hour after injecting *E. coli* suspended in the LB medium and positioned at the same level to track the progress of the spectra over time. The results indicate a gradual rise in the intensity of the band from 1050 to 1800 cm^−1^. This is probably due to the adhesion and accumulation of the bacteria on the Au surface. Although it seems that this was the band of interest, the reference and final spectra had been graphed in Figure 13 considering the band from 500 to 1000 cm^−1^. Figure 13 shows four peak shifts at 665, 780, 852, and 878 cm^−1^. The Raman peaks at 665, 780, and 1340 cm^−1^ are associated with expected components of residues of DNA [10,46].

**Figure 14:**
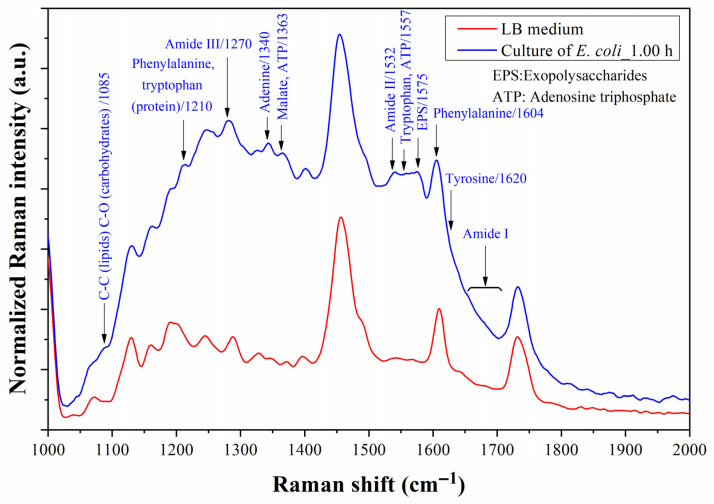
Raman spectra in the 1000–2000 cm^−1^ shift band of the microfluidic prototype measured with LB medium before testing and *E. coli* culture after 1 h of testing. The assigned Raman peaks correspond to the molecular vibrations of functional groups of *E. coli*.

Figure 14 shows a cocktail of Raman peaks related to amides and amino acids after one hour of testing. The amides II and III appear at 1532 and 1270 cm^−1^, respectively, while amide I is revealed in the band from 1660 to 1700 cm^−1^ [10,47,48,49]. These Raman shift assignments correspond to the protein structure of the outer membrane of bacteria, which increasingly adheres better to the Au surface. The Raman shifts at 1085 and 1575 cm^−1^ also corroborate well with the results of molecular vibrations of chains of C-C and C-O bonds of lipids, carbohydrates, and polysaccharides in the outer membrane and surface of the bacteria [47]. The primary three amino acids, products of the metabolic activity of bacteria, namely phenylalanine (Phe), tryptophan (Trp), and tyrosine (Tyr), were detected at 852 (Tyr), 878 (Trp), 1210 (Phe and Trp), 1557 (Trp), and 1620 (Tyr) cm^−1^ [10,47,48,49]. The buried Tyr at 852 cm^−1^ is related to tyrosine located within lipid sections of the membrane. A slight shift to the left of the Raman peak at 1604 cm^−1^ was associated with the presence of Phe. As additional data, Nguyen et al. associated the peaks at 1360 cm^−1^ and within the band from 1540 to 1560 cm^−1^ with the presence of adenosine triphosphate (ATP), which is also expected, given its participation in the activity of exopolysaccharides, flagella, and fimbria, among others, of the bacteria [50].

**Figure 15:**
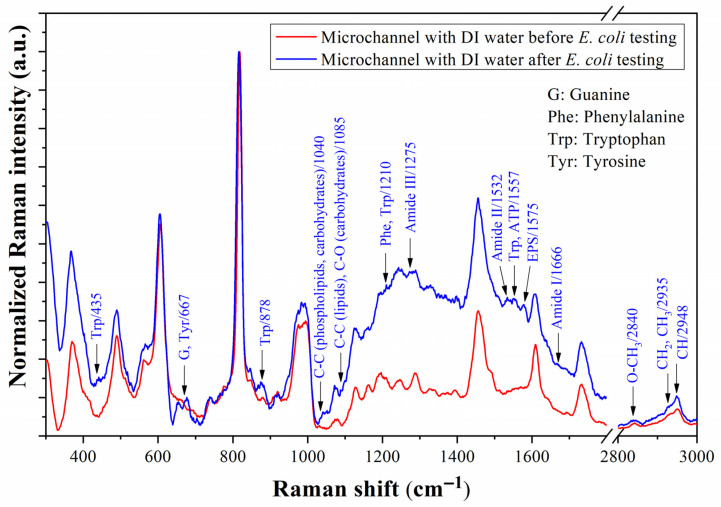
Raman spectra of the microfluidic prototype with deionized (DI) water before and after injecting *E. coli* culture into the microchannel. The assigned Raman peaks correspond to the molecular vibration of functional groups of *E. coli*.

Following the procedure described in Section 3.4, Figure 15 shows the spectra obtained from Raman measurement of the prototype with DI water in the microchannel before and after *E. coli* testing. According to these spectra, the residues of DNA were removed from the microchannel, and only the Raman peaks associated with bacterial adhesion to the Au surface were detected (see the previous paragraph). The band from 1530 to 1580 cm^−1^ is conserved after removing the *E. coli* culture in the LB medium. In addition, the Raman peaks at 435 and 1040 cm^−1^ related to tryptophan and the C-C chains of phospholipids and carbohydrates appear [10,51]. The conservation of the peaks assigned to Trp reveals that it is an amino acid characteristic of this strain of *E. coli* in the LB culture medium that is used in this proof.

## 5. Discussion

Given the polymeric nature of the microfluidic prototype, some Raman spectral bands of the biological compounds overlap with those of the PMMA substrate and the FOP film. However, a pair of bands in the ranges of 1530–1590 cm^−1^ and 1630–1700 cm^−1^ was available for the detection of the *E. coli* bacteria. The detection in these bands was possible due to the SERS effect and the measurement parameters. The 3D simulation results showed that EFs of approximately 10^11^ were achieved at the middle point of the nanogaps between Au nano-hemispheres. Similar results have been published by other groups [52,53,54]. Through 3D simulations using the finite element method, Yan et al. found that the electric field in the gap of a dimer is concentrated in the middle between the two nanoparticles and decreases progressively from this point to the surface of the nanoparticles [52]. A similar pattern was found by Chien et al. experimenting with regular arrays of silver-coated polystyrene nanospheres [53]. They also found a hot spot with a SERS EF of approximately 10^8^. In the same line, Zhu et al. achieved an average SERS EF of 10^11^ using substrates with hemispherical-type 3D nanostructures [54]. This improvement in SERS EF using nano-hemispheres, compared to nanospheres, was also reported by Wang et al. [55]. As the work of Solís et al. demonstrates, the EF is dependent not only on the size, shape, and distance of the nanoparticles, and the excitation wavelength, but also on the number of nanoparticles making up the arrangement, which they called “realistic configurations” [56]. They found that arrays of hundreds of nanoparticles result in higher EFs compared to dimers. Similar results were published by Knorr et al., finding, among the nanostructures analyzed, the best SERS performance with hemispheres [57]. At this point, it is worth mentioning that this work focused on evaluating the proposed microfluidic prototype. Therefore, the simulations were configured according to the experimental setup, leaving out of scope the analyses related to variations in the angle of incidence or laser light parameters such as wavelength or polarization, which are of interest for future advances and have been addressed in [52,55,56,57,58].

The spectrophotometer’s internal optical system was configured based on the application and factors such as power, wavelength, and radiation time. The wavelength of 785 nm was selected because it generates weaker fluorescence, maintaining good performance in molecular excitation. A power of 90–100 mW was required because the Au layer is in the excitation path, which reduces the intensity of the radiation in the detecting zone. Consequently, the exposure time was adjusted to 1.5 min, avoiding damage to the bacteria by the delivered dose of ~9 J [59]. Regarding the materials, the optical properties of the PMMA, FOP, and Au layers are not an obstacle to the propagation of the radiated waves; thus, the laser excitation reaches the microchannel, and the response signals return to the microscope.

We believe that the success in detecting the cysteamine monolayer and *E. coli* bacteria employing the SERS effect with this microfluidic prototype could be based on the conception of the prototype, including how it is coupled to the measurement equipment. The metal layer is right on the top wall of the microchannel; then, it interacts first with the laser radiation than any other component within the microchannel. For this reason, any compound in contact with the Au surface is reported in the Raman spectrum of the prototype with higher intensity. This statement is supported by the results of the simulations and experiments (Figure 9, Figure 10, Figure 11, Figure 12, Figure 13, Figure 14 and Figure 15). Regarding the treatment of the Au layer with HF solution, according to the results in Figure 7, this treatment opens windows through the Au thin film; accordingly, the laser radiation reaches the other side of the metal layer (i.e., the microchannel in the case of the prototype). Although the spontaneous formation of nanoparticles from nanometric Au films deposited on FOP surfaces has been reported by Hernández-Cruz et al. [23], the HF treatment accelerates the Au layer stabilization, achieving the final state in less time (the spontaneous formation of nanoparticles takes more than 10 days).

The main interest of this work was the characterization of the prototype and the corroboration of its performance as a polymer-based SERS platform. Throughout this work, it was possible to demonstrate that the manufacturing process is viable and that even products of the metabolic activity of the bacteria could be detected. *E. coli* detection was achieved in just 1 h, as in previous reports [13,14], with small sample volumes (10 µL minimum), without the need for labels and undesirable environmental effects. Specificity plays a predominant role. To achieve this, the metal surface must be functionalized with bioreceptors [13]. However, each layer of functionalization ends up separating the metal surface from the microorganisms, with which the amplitudes of the signals associated with the microorganisms are reduced (lesser SERS effect), and thus the sensitivity of the measurement [60]. Achieving the functionalization of the surface while minimally compromising the measurement is a challenge. On the other hand, reaching a broader spectrum of users requires eliminating the dependence on the Raman microscope. In this case, it is undoubtedly possible to advance its development by incorporating a 785 nm laser diode and a multispectral sensor operating in the visible range to capture the resulting radiation. This would give rise to a more compact version of the prototype.

Currently, the progress in creating a sensing platform from a detection prototype is still ongoing. This involves conducting tests with varying concentrations of *E. coli* to determine important metrics such as sensitivity, limit of detection, and accuracy, among others.

## 6. Conclusions

A microfluidic prototype for monitoring surface processes and detecting *E. coli* using SERS was proposed, simulated, fabricated, and tested. The results of layer-by-layer characterization demonstrated that it is possible to excite the surface inside the microchannel and obtain a response from the molecules deposited on the gold surface that form the upper wall of the microchannel. A treatment in HF solution was required to stabilize the Au layer. Although the Raman shifts of the polymeric materials overlap with some of the biological compounds, the detection of the *E. coli* bacteria attached to the Au layer was achieved through the bands of 1530–1590 cm^−1^ and 1630–1700 cm^−1^. These results open an opportunity for the development of polymer-based SERS platforms for surface functionalization, microorganism detection, and studies on bacterial resistance to antibiotics.

## Figures and Tables

**Table 1:** Refractive indices and extinction coefficients of the materials at 785 nm of wavelength.

Material	Refractive Index	Extinction Coefficient
Air	1	0
PMMA ^a^	1.4860	1.2650 × 10^−7^
FOP ^b^	1.4522	0
Au ^c^	0.4708	4.9876
H_2_O ^d^	1.3284	1.5430 × 10^−7^

^a^ Value based on data measured by Zhang et al. [26]. ^b^ Value calculated using the Vogel model [27,28]. ^c^ Value supplied by F. Lemarchand in a private communication in 2013 (4.62 nm film; n, k 0.35–1.8 µm) and published on the RefractiveIndex.INFO website. ^d^ Value based on data measured by Kedenburg et al. [29].

**Table 2:** Average and maximum SERS enhancement factors (EFs, (|*E*|/|*E*_0_|)^4^) in planes.

Day	EF in xy Plane z = 5 nm		EF in xz Plane x = [−0.1, 0.1] nm z = [–25, 35] nm		EF in yz Plane y = [−0.1, 0.1] nm z = [−25, 35] nm	
	Average	Maximum	Average	Maximum	Average	Maximum
6	5.6195 × 10^9^	7.7637 × 10^11^	4.7876 × 10^7^	2.3894 × 10^9^	5.3242 × 10^7^	2.2011 × 10^9^
12	5.5212 × 10^8^	1.0411 × 10^11^	6.5086 × 10^6^	1.2200 × 10^8^	8.1982 × 10^6^	5.8675 × 10^8^
18	8.4126 × 10^7^	3.5902 × 10^10^	6.9237 × 10^5^	4.1541 × 10^7^	5.7364 × 10^6^	6.0196 × 10^8^
24	3.9312 × 10^7^	1.3120 × 10^10^	2.0354 × 10^4^	5.9255 × 10^5^	7.8220 × 10^6^	2.3317 × 10^8^

## Data Availability

The measurement data and simulation files will be provided upon request to the corresponding author by email.
